# Supplementary material for: Spin-orbital Jahn-Teller bipolarons
Source: Nat Commun. 2024 Mar 18;15:2429. doi: 10.1038/s41467-024-46621-0 (PMC11258125; doi:10.1038/s41467-024-46621-0)
Supplement: Supplementary file 1 — Supplementary Information [file 41467_2024_46621_MOESM1_ESM.pdf]

Supplementary Information for Spin-orbital Jahn-Teller bipolarons

Lorenzo Celiberti[1,2], Dario Fiore Mosca[1,3,4], Giuseppe Allodi[5], Leonid V. Pourovskii[3,4], Anna Tassetti[2], Paola Caterina Forino[2], Rong Cong[6], Erick Garcia[6], Phuong M. Tran[7], Roberto De Renzi[5], Patrick M. Woodward[7], Vesna F. Mitrović[6], Samuele Sanna[2], and Cesare Franchini[1,2,*]

[1] *University of Vienna, Faculty of Physics and Center for Computational Materials Science, Vienna, Austria*
[2] *Department of Physics and Astronomy 'Augusto Righi', Alma Mater Studiorum - Università di Bologna, Bologna, 40127 Italy*
[3] *Centre de Physique Théorique, Ecole polytechnique, CNRS, Institut Polytechnique de Paris, 91128 Palaiseau Cedex, France*
[4] *Collège de France, Université PSL, 11 place Marcelin Berthelot, 75005 Paris, France*
[5] *Department of Mathematical, Physical and Computer Sciences, University of Parma, 43124 Parma, Italy*
[6] *Department of Physics, Brown University, Providence, Rhode Island 02912, USA*
[7] *Department of Chemistry and Biochemistry, The Ohio State University, Columbus, Ohio 43210, USA*

[*] *e-mail: cesare.franchini@univie.ac.at*

Supplementary Note 1: DFT polaronic levels

The polaronic wavefunctions were calculated at the DFT level using the spinorial projected localised orbitals (PLOs) defined by Fiore Mosca *et al.* [1] and Schüler *et al.* [2] within the projector augmented wave (PAW) scheme. Specifically, an effective Hamiltonian $H_{nn'}$ was obtained by projecting the Kohn-Sham energy levels $\epsilon_m(\mathbf{k})$ onto the subspace generated by the angular momentum $l = 2$ wavefunctions centered at the polaronic site and radial part obtained from the PAW projectors, as implemented in the TRIQS dftTOOLs package [3]. The Hamiltonian was projected onto the local correlated space as obtained from the following equation, in which $H_{mm'}(\mathbf{k}) = \epsilon_m(\mathbf{k})\delta_{mm'}$

$$H^{\alpha\beta}_{nn'} = \sum_{\mathbf{k}} \tilde{P}^{\mathcal{C}\beta}_{nm'}(\mathbf{k}) H_{mm'}(\mathbf{k}) \big(\tilde{P}^{\mathcal{C}\alpha}_{mn}(\mathbf{k})\big)^* \ , \tag{1}$$

where

$$P^{\alpha}_{\mathcal{C},L}(\mathbf{k}) = \sum_i \langle \chi_L | \phi_i \rangle \langle \tilde{p}_i | \tilde{\Psi}^{\alpha}_{\mathbf{k}} \rangle \ , \tag{2}$$

are the spinorial PLOs written in the PAW formalism [1].

The projectors were calculated after convergence of the DFT self-consistent cycle within an energy window of $2.25 \div 9.75$ eV with respect to the Fermi level, in order to include all d levels of the polaronic Os site. At this stage, the Hamiltonian was given in the $|d_a, s_z\rangle$ basis, where $a = \{xy, xz, yz, z^2, x^2 - y^2\}$ and $s_z = \pm 1/2$. In terms of l^2 and l_z common eigenstates they are given by

$$\begin{aligned}
|d_{xy}, s_z\rangle &= -\frac{i}{\sqrt{2}} \left(|2, s_z\rangle - |-2, s_z\rangle\right) \\
|d_{xz}, s_z\rangle &= -\frac{1}{\sqrt{2}} \left(|1, s_z\rangle - |-1, s_z\rangle\right) \\
|d_{yz}, s_z\rangle &= \frac{i}{\sqrt{2}} \left(|1, s_z\rangle + |-1, s_z\rangle\right) \\
|d_{z^2}, s_z\rangle &= |0, s_z\rangle \\
\left|d_{x^2-y^2}, s_z\right\rangle &= \frac{1}{\sqrt{2}} \left(|2, s_z\rangle + |-2, s_z\rangle\right)
\end{aligned} \tag{3}$$

The cubic harmonics $|d_a\rangle$ are defined with respect to the VASP internal reference frame, so that in our $\sqrt{2}a \times \sqrt{2}a \times a$ supercell the x, y and z axes correspond respectively to the [110], [1$\bar{1}$0] and [001] crystallographic axes. In order to have real harmonics defined in the reference frame of the OsO_6 octahedron we rotate the orbital $l = 2$ and spin $s = 1/2$ subspaces using Wigner $D^j_{mm'}(\alpha, \beta, \gamma)$ matrices, defined as

$$D^j_{mm'}(\alpha, \beta, \gamma) = \langle j, m' |\, e^{-i\alpha J_z} e^{-i\beta J_y} e^{-i\gamma J_z} \,| j, m\rangle \tag{4}$$

where α, β and γ are Euler angles describing the reference frame rotation and J_y and J_z are cartesian coordinates of the angular momentum operator $\mathbf{J}$. In our case the rotation is described by the angles $\alpha = 0$, $\beta = 0$, $\gamma = \pi/4$.

Since the crystal field interaction (~ 5 eV) is much larger than SO interaction (~ 0.3 eV) in BNOO, we can separate t_{2g} and e_g orbitals and consider only the former in our analysis. In the octahedron reference frame they are defined as:

$$\begin{aligned}
\left|t^1_{2g}\right\rangle &= -\frac{1}{\sqrt{2}} (|d_{xz}\rangle + i\,|d_{yz}\rangle) \\
\left|t^0_{2g}\right\rangle &= |d_{xy}\rangle \\
\left|t^{-1}_{2g}\right\rangle &= \frac{1}{\sqrt{2}} (|d_{xz}\rangle - i\,|d_{yz}\rangle)
\end{aligned} \tag{5}$$

One can easily show that the projection of the angular momentum operator $\mathbf{L}$ onto the t_{2g} orbitals give

$$P_{t_{2g}} \mathbf{L} P_{t_{2g}} = -\mathbf{l} \tag{6}$$

where $\mathbf{l}$ is an effective angular momentum operator with $l = 1$.

At this point, we employ Clebsch-Gordan coefficients to construct $J_{\text{eff}} = 3/2$ and $J_{\text{eff}} = 1/2$ states out of the t_{2g} ones. To conclude, we rotated again our basis with Wigner D-matrices to bring the angular momentum quantization axis along the direction of magnetisation, which lies in the (110) plane. The occupation matrix thus obtained is pictorially reproduced in Supplementary Fig. 1 and numerically in Supplementary Tab. 1.

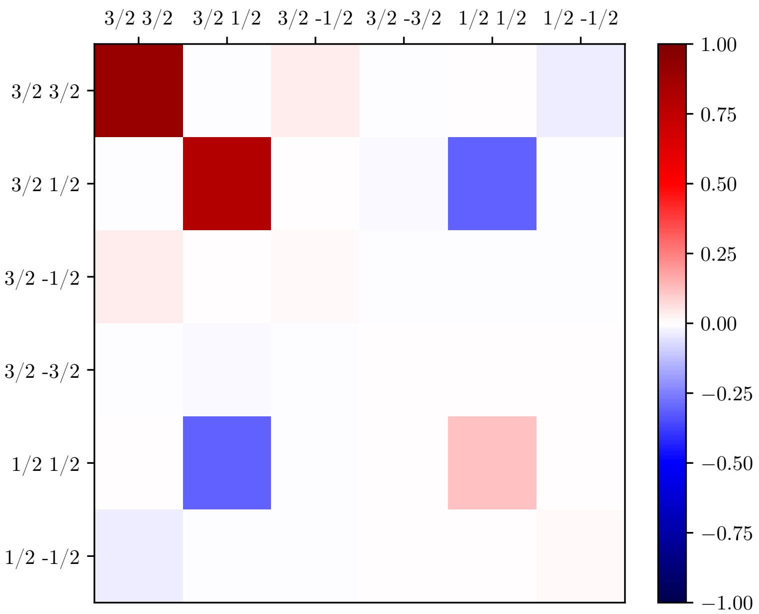

Supplementary Figure 1: Diagram of the polaronic occupation matrix as calculated from DFT non-collinear projectors. Axes are labelled according to (j, j_z) values. Colors represent the value of the occupation matrix reported in Supplementary Tab. 1. Dark red on the diagonal corresponds to level occupation equal to one. The diagram shows that the DFT polaron occupies $J_{\text{eff}} = 3/2$ states $|3/2, 3/2\rangle$ and $|3/2, 1/2\rangle$ with the latter state slightly mixed with the $J_{\text{eff}} = 1/2$ $|1/2, 1/2\rangle$.

	3/2, 3/2	3/2, 1/2	3/2, -1/2	3/2, -3/2	1/2, 1/2	1/2, -1/2
3/2, 3/2	0.90	0.00	0.04	0.00	0.00	-0.04
3/2, 1/2	0.00	0.80	0.00	-0.01	-0.31	0.00
3/2, -1/2	0.04	0.00	0.01	0.00	0.00	0.00
3/2, -3/2	0.00	-0.01	0.00	0.00	0.00	0.00
1/2, 1/2	0.00	-0.31	0.00	0.00	0.12	0.00
1/2, -1/2	-0.04	0.00	0.00	0.00	0.00	0.01

Supplementary Table 1: Polaronic occupation matrix calculated from DFT non-collinear projectors. Column and row labels indicate the matrix components on the $J_{\text{eff}} = 3/2$ and $J_{\text{eff}} = 1/2$ levels.

Supplementary Note 2: HI polaronic levels

Charge self-consistent DFT+DMFT calculations (see Methods section of the main text) predicted a multiplet structure of the two-electron levels at the polaronic site, as depicted in Supplementary Fig. 2. In particular, we can distinguish a low lying quintuplet, a triplet about 0.4 eV above the ground state multiplet and a singlet. The next excited state lies about 1 eV above those represented in Supplementary Fig. 2. By looking at the wavefunctions of the ground state quintuplet, we can see that they are well represented by $J_{\mathrm{eff}} = 2$ spin-orbital states (see Supplementary Tab. 2).

The one-electron spectral density was also calculated for the d^1 and d^2 states and is reported in Supplementary Fig. 3. The DFT+HI polaronic states appear in the band gap just above the valence d^1 states.

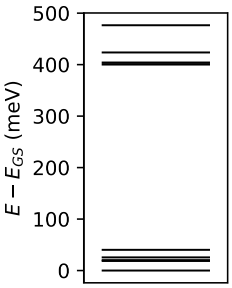

Supplementary Figure 2: Lowest two-electron energy levels.

Energy (meV)	$J = 2$ multiplet eigenstate
0.0	$+0.999\,\lvert 2;0\rangle - (0.031 - 0.003i)\,\lvert 2;2\rangle - (0.031 + 0.003i)\,\lvert 2;-2\rangle$
18.6	$+0.707\,\lvert 2;-1\rangle - (0.701 - 0.094i)\,\lvert 2;1\rangle$
20.3	$+0.707\,\lvert 2;1\rangle + (0.701 + 0.094i)\,\lvert 2;-1\rangle$
25.6	$+0.706\,\lvert 2;2\rangle + 0.706\,\lvert 2;-2\rangle + 0.044\,\lvert 2;0\rangle$
40.2	$+0.707\,\lvert 2;-2\rangle - 0.707\,\lvert 2;2\rangle + (0.000 - 0.004i)\,\lvert 2;0\rangle$

Supplementary Table 2: Multiplet eigenstates of the first five levels. The first row of the table indicates that the polaronic ground state has a spin-orbital character with $J_{\mathrm{eff}} = 2$.

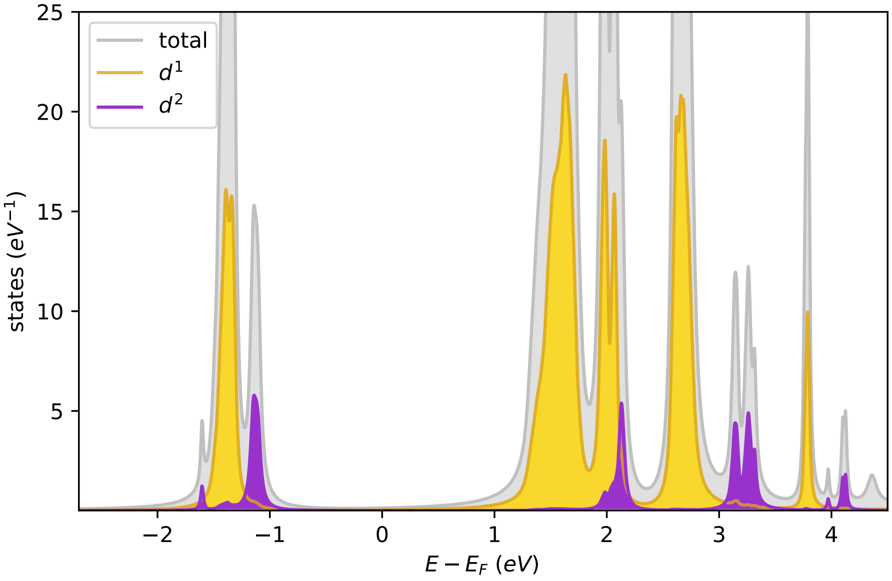

Supplementary Figure 3: Single particle spectral density. Contributions of the d^1 and d^2 sites are highlighted respectively in yellow and violet. The violet polaronic peak lies just above the conduction band, around −1 eV.

Supplementary Note 3: Polaron formation energy vs JT modes

The JT normal coordinates used in our analysis are defined as linear combinations of the Cartesian displacements X_i, Y_i, and Z_i of the oxygen atoms located at the corners of the distorted octahedron structure (see Supplementary Fig. 4). These displacements are defined with respect to their corresponding positions in a reference undistorted structure, denoted by $x_i^{(0)}, y_i^{(0)}, z_i^{(0)}$. Starting from X_i, Y_i, and Z_i, JT normal coordinates Q_i can be defined that transform according to the irreducible representations of the octahedral group O_h [4], as represented in Supplementary Tab. 3.

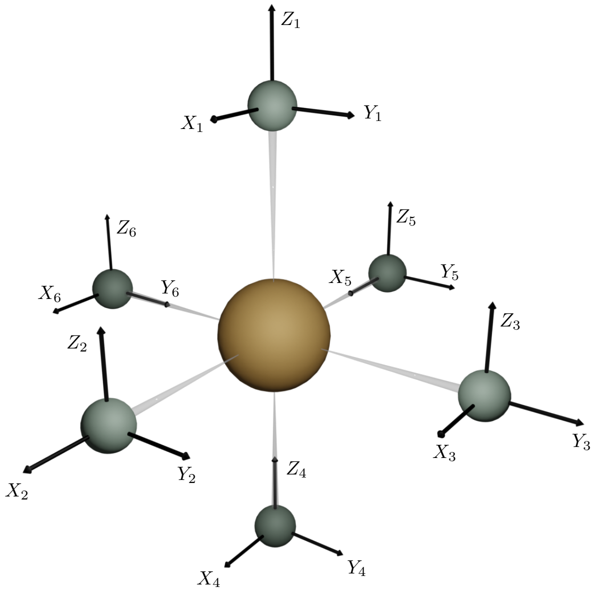

Supplementary Figure 4: Graphical representation of the cartesian distortions X_i, Y_i and Z_i.

A_{1g}	Q_1	$(X_2 - X_5 + Y_3 - Y_6 + Z_1 - Z_4)/\sqrt{6}$
E_g	Q_2	$(X_2 - X_5 - Y_3 + Y_6)/2$
	Q_3	$(2Z_1 - 2Z_4 - X_2 + X_5 - Y_3 + Y_6)/2\sqrt{3}$
	Q_{yz}	$(Z_3 - Z_6 + Y_1 - Y_4)/2$
T_{2g}	Q_{xz}	$(X_1 - X_4 + Z_2 - Z_5)/2$
	Q_{xy}	$(Y_2 - Y_5 + X_3 - X_6)/2$
	Q'_x	$(X_1 + X_3 + X_4 + X_6)/2$
T'_{1u}	Q'_y	$(Y_1 + Y_2 + Y_4 + Y_5)/2$
	Q'_z	$(Z_1 + Z_3 + Z_4 + Z_6)/2$
	Q''_x	$(X_2 + X_5)/\sqrt{2}$
T''_{1u}	Q''_y	$(Y_3 + Y_6)/\sqrt{2}$
	Q''_z	$(Z_1 + Z_4)/\sqrt{2}$
	Q'_{xy}	$(X_3 + X_6 - X_1 - X_4)/2$
T_{1u}	Q'_{yz}	$(Y_1 + Y_4 - Y_2 - Y_5)/2$
	Q'_{xz}	$(Z_2 + Z_5 - Z_3 - Z_6)/2$
	$Q_{r,yz}$	$(Z_3 - Z_6 + Y_4 - Y_1)/2$
rot.	$Q_{r,xz}$	$(X_1 - X_4 - Z_2 + Z_5)/2$
	$Q_{r,xy}$	$(Y_2 - Y_5 - X_3 + X_6)/2$

Supplementary Table 3: Definition of the octahedron vibration coordinates Q_i as a function of the cartesian distortions X_i, Y_i and Z_i.

We used the BNOO cubic structure with full $Fm\bar{3}m$ symmetry [5] as a reference. Cartesian directions X_i, Y_i and Z_i correspond to crystallographic axes [100], [010] and [001] respectively. In all our calculations, we find only four modes to be different from zero: Q_1, Q_2, Q_3, and Q_{xy}. Their values for both the pristine BNOO with cAFM ordering and the polaronic site are reported in Supplementary Tab. 4. The T_{2g} mode Q_{xy} is not mentioned in previous studies on pristine BNOO [6].

	Q_1 (Å)	Q_2 (Å)	Q_3 (Å)	Q_{xy} (Å)
pristine	0.057	0.017	-0.005	0.019
polaron	0.138	-0.011	0.014	-0.010

Supplementary Table 4: Non-zero deformation modes for an OsO_6 octahedron in the pristine material (first row) and for a polaronic one (second row).

We provide in the following the analysis of the effect of non-zero modes on the polaronic formation energy and their relation to SO effects. First, we observe how polaron energy E_{pol} changes as a function of the isotropic mode Q_1 and the E_g modes Q_2 and Q_3. In particular, starting from the distortions obtained for the polaronic ground state, we calculate all the Q_i of Supplementary Tab. 3. Then, we fix all Q_i but the one we want to study and invert the transformation between Q_i and Cartesian displacements to generate the new structure, where the polaron has one mode changed and all others are left unchanged. By fixing the position of the polaronic OsO_6 octahedron and letting all other ions to relax, we calculate the polaron energy always with respect to the same delocalised configuration. In this way, we obtain the parabolas reported in Supplementary Fig. 5. Here, the normalised distortions $\tilde{Q}_i$ are defined as the ratio of the polaronic and pristine distortion modes. In this way we can easily estimate the energy gained by the polaron by change one mode Q_i with respect to its value in the pristine case $\tilde{Q}_i = 1$. The same calculations for Q_{xy} show an energy gain of the same order of magnitude of those obtained for the E_g modes.

As a second step, we explore the relation between the JT modes and SO intensity c_λ. The A_{1g} mode Q_1, corresponding to an isotropic expansion of the octahedron, stays constant throughout the range of SO intensity (see Supplementary Fig. 6(a)). For the E_g and T_{2g} modes, two different behaviours could be distinguished. The

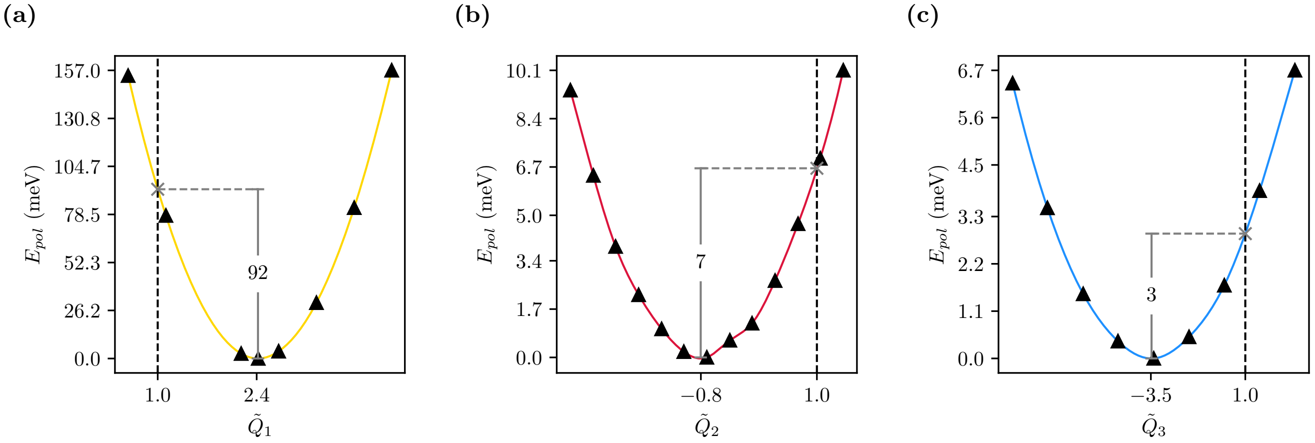

Supplementary Figure 5: Polaron energy E_{pol} as a function of the normalised **(a)** $\tilde{Q}_1$, **(b)** $\tilde{Q}_2$ and **(c)** $\tilde{Q}_3$ modes for $c_\lambda = 1$. Dashed lines correspond to the pristine values of $\tilde{Q}_i$. Solid grey lines indicate the lattice energy gain given by a polaronic distortion with respect to the pristine one. The breathing-out mode $\tilde{Q}_1$ presents the major contribution amounting to $\approx 67\%$ of the polaron energy.

tetragonal elongation in the [001] direction Q_3 decreases with increasing c_λ, as already mentioned in the main text and also found by Streltsov and Khomskii [7]. On the other hand, Q_2 and Q_{xy} remain constant above $c_\lambda \simeq 0.2$ and rapidly go to zero for $c_\lambda < 0.2$, as represented in Supplementary Fig. 6(c-d). However, previous studies predicted exactly the opposite for a d^2 JT impurity: all E_g and T_{2g} distortions should decrease in amplitude for increasing c_λ [7, 8]. Moreover, the different behaviours of Q_2 and Q_3 suggest Therefore, we supposed that the Q_2 and Q_{xy} distortions have a different origin than the Q_3 one.

To prove this assumption, we estimate the JT energy E_{JT} of the polaronic octahedron as a function of c_λ in a quasimolecular approximation [4, 9]. In particular, we calculate all the Q_i from the polaronic ground state structure, fix all of them but a chosen one, and invert the transformation of Supplementary Tab. 3 between Cartesian displacements and generalised modes Q_i to generate the distorted structures needed to construct the ionic potential energy surface. For each distorted structure, total energies are calculated with the occupation matrix constrained to that of the polaronic ground state [10]. The parabolas $E(Q_i)$ obtained in this way have been fitted to estimate the JT energy gain E_{JT} as the difference between the energy at the minimum and that corresponding to the structure with the varying Q_i equal to zero:

$$E_{\mathrm{JT}} = E(Q_i^{pol}) - E(Q_i = 0) \tag{7}$$

where Q_i^{pol} is the value of the distortion for the polaronic ground state. The results are shown in Fig. 3 of the main text. While E_{JT} was one order of magnitude more negative for the tetragonal distortion Q_3 at weak SO coupling, for Q_2 and Q_{xy} the JT energy gain remains smaller than 1 meV throughout the explored c_λ range. We therefore assume that their contribution to the polaron stability is negligible compared to the Q_3 one.

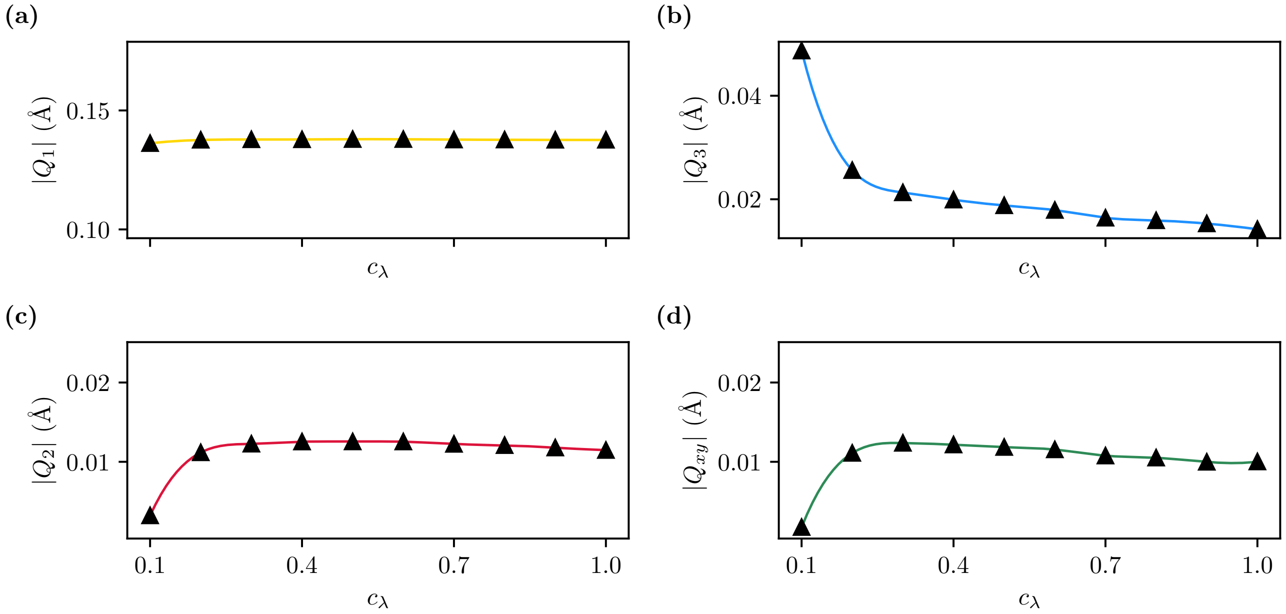

Supplementary Figure 6: Non-zero deformation modes behaviour as a function of SO coupling strength c_λ. **(a)** the breathing-out mode Q_1 shows no SO dependence. **(b)** the tetragonal mode Q_3 is quenched by SOC. **(c-d)** the orthorhombic mode Q_2 and the trigonal one Q_{xy} are completely suppressed at very low SO and show a plateau for $c_\lambda \gtrsim 0.2$.

Supplementary Note 4: DOS for different Ca concentrations

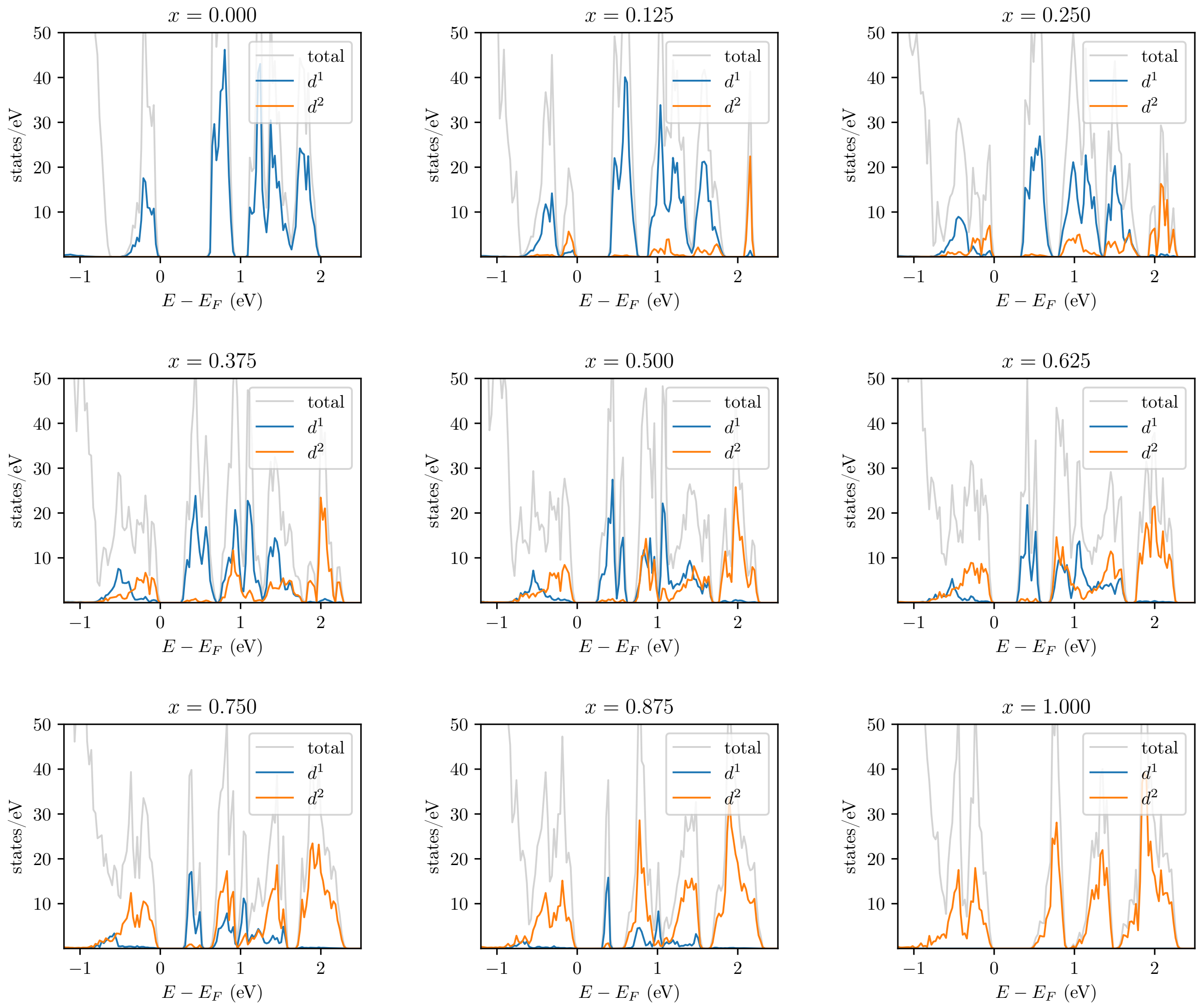

Supplementary Figure 7: DOS projected onto the d-orbitals of Os d^1 (blue) and d^2 (orange) sites at different Ca concentration x. The total DOS is shown in light grey.

Supplementary Note 5: NMR anomalous peak fit results

We have fitted the NMR $1/T_1$ anomalous peak using the BPP-like model of Eq. (1). The results are reported in Supplementary Tab. 5.

x(%)	T_a (K)	E_a (meV)	τ_0 (ps)	$\Delta^2 \times 10^9$ (s^{-2})
12.5	855 ± 26	74 ± 2	0.7 ± 0.2	28.0 ± 0.8
25.0	788 ± 21	68 ± 2	2.5 ± 0.4	27.5 ± 0.6
37.5	953 ± 38	82 ± 3	0.7 ± 0.2	97 ± 3
50.0	931 ± 24	80 ± 2	0.8 ± 0.2	129 ± 3
75.0	928 ± 23	80 ± 2	0.9 ± 0.2	143 ± 3
90.0	857 ± 24	77 ± 2	1.1 ± 0.2	97 ± 2

Supplementary Table 5: NMR anomalous peak fitting results

Supplementary Note 6: Determination of the relaxation times T_1 and T_2

The measure of T_1 is extracted by using the standard saturation recovery method with echo detection. In these conditions the nuclear spin transitions can be fully saturated but the detection reveals only the central transition. In our sample $Ba_2Ca_{0.125}Na_{0.875}OsO_6$, the NMR signal amplitude as a function of the repetition delay time t can be fitted to a stretched exponential function:

$$M(t) = M_0\left(1 - exp\left(-\left(\frac{t}{T_1}\right)^{\beta_1}\right)\right) \tag{8}$$

where the common interpretation of $\beta < 1$ is, in terms of the global relaxation, that the sample contains many independently relaxing species, resulting in the sum of different exponential decays. This is expected in systems with considerable electronic inhomogeneity, giving rise to a broad distribution of correlation times [11–13].

The stretched behaviour is often observed in complex transition metal oxides. It typically reflects the presence of a non trivial distribution of relaxation rates due to local electronic inhomogeneities, which give rise to a site dependent magnetic or electric coupling.

A single exponential decay is clearly corresponding to $\beta = 1$, while $\beta = 0.5$ is typical of a fully disordered system, *i.e.* an intrinsic heterogeneity of phases, which can be described with a multi-exponential factor.

Supplementary Fig. 8 shows the experimental behaviour with the fit line of Supplementary Eq. (8), for the normalized amplitude as a function of the delay time, for representative temperatures.

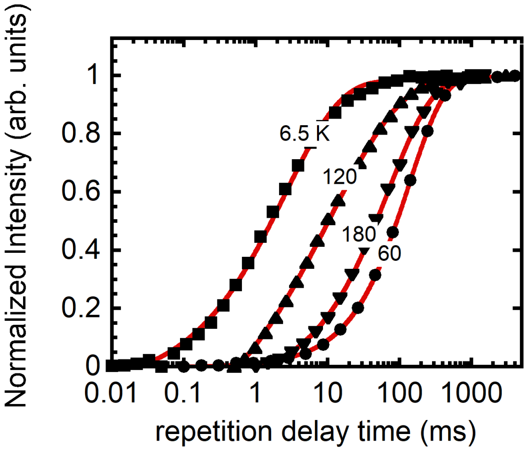

Supplementary Figure 8: Plot of the normalized amplitude signal as a function of the delay for the T_1 relaxation of the $Ba_2Na_{0.125}Ca_{0.875}OsO_6$ sample with the fit (solid line) to Supplementary Eq. (8).

The relaxation rate $1/T_1$ if displayed in the main text and the β_1 coefficient as a function of temperature in the whole range are reported in Supplementary Fig. 10 (squares). Notice that the stretching coefficient is reduced to $\beta = 0.5$ at the relaxation rate's peak, which indicates that the electronic disorder reaches its maximum when cooling towards the crossover between the dynamic and the static regime on the NMR time scale, but it approaches a single exponential decay with $\beta = 1$ elsewhere. The single relaxation rate is expected also for quadrupolar nuclei ($I > 1/2$) in a cubic crystal since the static quadrupolar interaction vanishes and spin-spin interactions maintain a spin temperature [14, 15]. Indeed, these conditions are satisfied throughout the reported temperature range, where a single NMR line is observed, with linewidth below 30 kHz [16].

The measurements for the determination of the T_2 transverse relaxation time have been performed by using a modified $\pi/2$- $\pi/2$ Hanh echo sequence).

The data has been fitted to a stretched exponential fit function, analogous to the one used for the T_1 longitudinal relaxation case in Supplementary Eq. (8), in the form:

$$M(2\tau) = M_0\ exp\left(-\left(\frac{2\tau}{T_2}\right)^{\beta_2}\right), \tag{9}$$

with β ranging from 0.5 to 2 reflecting a more dynamical disordered or more Gaussian character, respectively.

In Supplementary Fig. 9 is represented the fit function, expressed in the Supplementary Eq. (9), for the normalized amplitude as a function of the echo delay time, for representative temperatures.

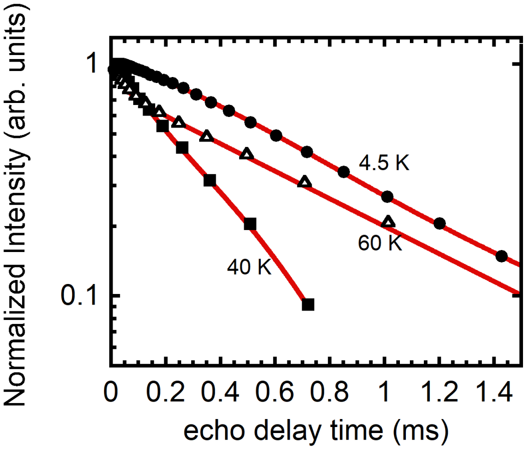

Supplementary Figure 9: Plot of the normalized amplitude signal as a function of the echo delay time for the T_2 relaxation of the $Ba_2Na_{0.125}Ca_{0.875}OsO_6$ sample, for selected temperatures with the fit (solid line) to Supplementary Eq. (9).

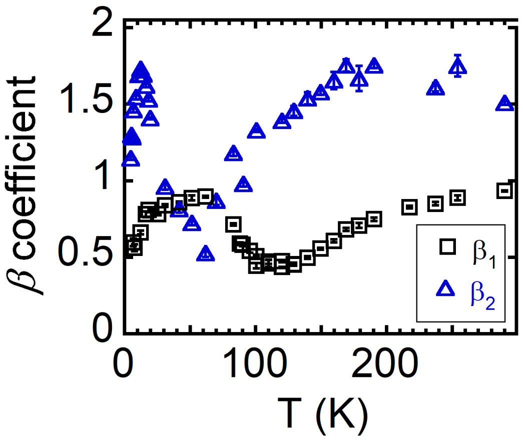

Supplementary Figure 10: Temperature dependence of the β_i exponent for $i = 1, 2$ of Supplementary Eq. (8) and Supplementary Eq. (9), respectively.

The relaxation rate $1/T_2$ if displayed in the main text and the β_2 coefficient as a function of temperature in the whole range are reported in Supplementary Fig. 10 (triangles).

The temperature dependence of the β parameter, both from T_1 and T_2, reflects the system dynamics. We can observe that local minima of β coefficients correspond to the peaks maxima of $1/T_1$ and $1/T_2$. This is expected in systems with a high electronic inhomogeneity which gives rise to a unresolved large distribution of correlation times.

Supplementary Note 7: Muon Spin Rotation

Muon spin relaxation measurements have been performed at the GPS instrument at the Paul Scherrer Institute (Switzerland) in both zero field (ZF), μ_0H=0, and longitudinal field (LF) conditions, where the latter uses an external field μ_0H parallel to the initial muon spin polarization.

After the implantation into powder samples of a beam of completely polarized positively charged muons (spin S_μ=1/2), we study the time evolution of muon spin asymmetry which provides information on the spatial distribution and dynamical fluctuations of the magnetic environment.

The μSR is a magnetic probe and allows to investigate local magnetic moments values, but is not sensitive to charge variations, because muons have no electric quadrupole moment and are typically insensitive to the static or dynamical effects of the EFG.

The implanted muons decay with a characteristic lifetime of 2.2 μs, emitting a positron preferentially along the direction of the muon spin. The positrons are detected and counted by a forward ($N_F(t)$) and backward detector ($N_B(t)$) as a function of time. The asymmetry function A(t) is given by

$$A(t) = \frac{N_B(t) - \alpha N_F(t)}{N_B(t) + \alpha N_F(t)}, \tag{10}$$

where α is a parameter determined experimentally from the geometry and efficiency of the μSR detectors. $A(t)$ is proportional to the muon spin polarization, and thus reveals information about the local magnetic field sensed by the muons. Examples of the muon asymmetry behavior are displayed in Supplementary Fig. 11. The zero field spectra are those previously reported in ref. [16] and accordingly in the magnetic phase each individual spectra was fitted to a sum of precessing and relaxing asymmetries given by

$$A(t) \quad = \left[A_1 e^{-\frac{\sigma_1^2 t^2}{2}} cos(2\pi\nu_1 t) + A_2 e^{-\frac{\sigma_2^2 t^2}{2}}\right] + A_\ell e^{-\lambda_\mu t} \tag{11}$$

The terms inside the brackets reflect the perpendicular component of the internal local field probed by the spin-polarized muons, the first term corresponds to the damped oscillatory muon precession about the local internal fields at frequencies ν_i, while the second reflects a more incoherent precession with a local field distribution given by σ, for a total of 2 different muon sites (accidentally the most general case accounts up to three inequivalent muon sites for other compositions of the same series [16]). The term outside the brackets reflects the longitudinal component characterized by the muon spin-lattice relaxation rate $\lambda_\mu \equiv 1/T_1^\mu$.

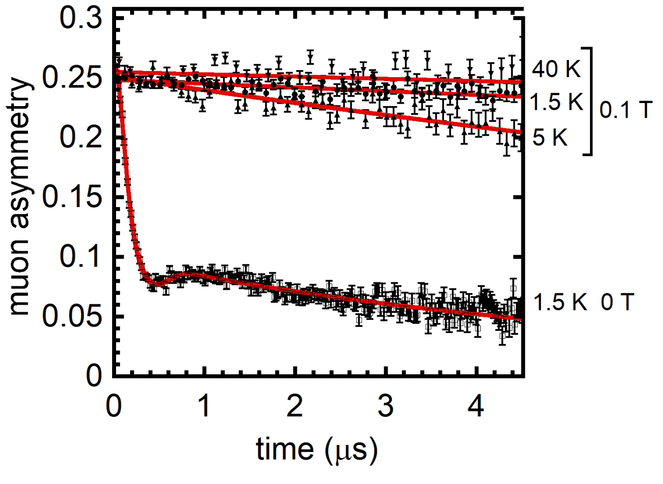

Supplementary Figure 11: Representative time dependence of the muon decay asymmetry with fit curves (lines) to Supplementary Eq. (11): open squares are for external zero field condition at 1.5 K and solid symbols are for longitudinal applied field of $\mu_0 H = 0.1$ T at selected temperatures (circle 1.5 K, up triangles 5 K, down triangles 40 K). Solid line are the best fit to Supplementary Eq. (11) and Supplementary Eq. (12) for ZF and LF, respectively

LF-μSR measurements have been performed as a function of temperature in order to apply a static field for two field of 10 and 100 mT being the latter much greater than the internal static field detected in the ordered phase

~ 10 mT as reported in Ref. [16]. In this condition the longitudinal amplitude (the tail) is fully recovered to the maximum amplitude already for few tens of mT also at the base temperature indicating a static character of the magnetic state. All the LF muon asymmetry data can be simply fitted to

$$A_\ell e^{-\lambda_\mu t} \tag{12}$$

Both the ZF and LF longitudinal rates are reported in Fig. 2(b) as a function of temperature. They clearly show a relaxation peak due to critical fluctuations when approaching the magnetic transition at $T_N \simeq 7$ K. No evidence of extra anomalous relaxation peak is detected at any temperature up to 300 K.

Supplementary Note 8: Modelling of spin-lattice relaxation rate

The spherical coordinate of the EFG appearing in Eq. (3) are given in cartesian coordinates by [17]

$$V_0 = \frac{1}{\sqrt{6}}\left[3V_{zz} - (V_{xx} + V_{yy} + V_{zz})\right] \tag{13a}$$

$$V_{\pm 1} = \mp\frac{1}{2}\left[V_{xz} + V_{zx} \pm i\,(V_{yz} + V_{zy})\right] \tag{13b}$$

$$V_{\pm 2} = \frac{1}{2}\left[V_{xx} - V_{yy} \pm i\,(V_{xy} + V_{yx})\right] \tag{13c}$$

where the lower indices indicate derivatives with respect to the corresponding cartesian coordinate. To estimate the EFG we used a point-charge model of the NaO_6 octahedron with a coulombic potential given by

$$V(\boldsymbol{r}) = q_{ox}\sum_{i=1}^{6}\frac{1}{|\boldsymbol{r} - \boldsymbol{R}_i|} \tag{14}$$

where q_{ox} is the formal charge of the oxagen ions and $\boldsymbol{R}_i$ is the position of the i-th oxygen ion with respect to the nucleus. For the oxygen labelling convention see Supplementary Fig. 4.

By combining Supplementary Eq. (13) and Supplementary Eq. (14) we can calculate the matrices $M^{(ij)}_{\alpha\beta}$ used in the derivation of the spin-lattice relaxation rate $1/T_1$ in the Methods section.

$$M^{(11)} = M^{(44)} = \frac{1}{R_0^8}\begin{pmatrix}3 & 0 & 0\\ 0 & 3 & 0\\ 0 & 0 & 9\end{pmatrix} \qquad M^{(22)} = M^{(55)} = \frac{1}{R_0^8}\begin{pmatrix}9 & 0 & 0\\ 0 & 3 & 0\\ 0 & 0 & 3\end{pmatrix} \qquad M^{(33)} = M^{(66)} = \frac{1}{R_0^8}\begin{pmatrix}3 & 0 & 0\\ 0 & 9 & 0\\ 0 & 0 & 3\end{pmatrix} \tag{15}$$

$$M^{(14)} = \frac{1}{R_0^8}\begin{pmatrix}-3 & 0 & 0\\ 0 & -3 & 0\\ 0 & 0 & -9\end{pmatrix} \qquad M^{(25)} = \frac{1}{R_0^8}\begin{pmatrix}-9 & 0 & 0\\ 0 & -3 & 0\\ 0 & 0 & -3\end{pmatrix} \qquad M^{(36)} = \frac{1}{R_0^8}\begin{pmatrix}-3 & 0 & 0\\ 0 & -9 & 0\\ 0 & 0 & -3\end{pmatrix} \tag{16}$$

$$M^{(12)} = -M^{(15)} = -M^{(42)} = M^{(45)} = \frac{1}{R_0^8}\begin{pmatrix}0 & 0 & 3\\ 0 & 0 & 0\\ -9/2 & 0 & 0\end{pmatrix} \tag{17}$$

$$M^{(13)} = -M^{(16)} = -M^{(43)} = M^{(46)} = \frac{1}{R_0^8}\begin{pmatrix}0 & 0 & 0\\ 0 & 0 & -3\\ 0 & -9/2 & 0\end{pmatrix} \tag{18}$$

$$M^{(23)} = -M^{(26)} = -M^{(53)} = M^{(56)} = \frac{1}{R_0^8}\begin{pmatrix}0 & -9/2 & 0\\ 3 & 0 & 0\\ 0 & 0 & 0\end{pmatrix} \tag{19}$$

References

[1]D. F. Mosca, H. Schnait, L. Celiberti, M. Aichhorn, and C. Franchini, *The Mott transition in the 5d*^{1} *compound* Ba_2NaOsO_6*: a DFT+DMFT study with PAW non-collinear projectors*, 2023, arXiv:2303.16560.

[2]M. Schüler, O. E. Peil, G. J. Kraberger, R. Pordzik, M. Marsman, G. Kresse, T. O. Wehling, and M. Aichhorn, "Charge self-consistent many-body corrections using optimized projected localized orbitals", Journal of Physics: Condensed Matter **30**, 475901 (2018).

[3]M. Aichhorn, L. Pourovskii, P. Seth, V. Vildosola, M. Zingl, O. E. Peil, X. Deng, J. Mravlje, G. J. Kraberger, C. Martins, M. Ferrero, and O. Parcollet, "TRIQS/DFTTools: A TRIQS application for ab initio calculations of correlated materials", Computer Physics Communications **204**, 200–208 (2016).

[4]I. Bersuker, *The Jahn-Teller Effect* (Cambridge University Press, Cambridge, 2006).

[5]K. E. Stitzer, M. D. Smith, and H.-C. zur Loye, "Crystal growth of Ba_2MOsO_6 (M=Li, Na) from reactive hydroxide fluxes", Solid State Sciences **4**, 311–316 (2002).

[6]D. Fiore Mosca, L. V. Pourovskii, B. H. Kim, P. Liu, S. Sanna, F. Boscherini, S. Khmelevskyi, and C. Franchini, "Interplay between multipolar spin interactions, Jahn-Teller effect, and electronic correlation in a $J_{\mathrm{eff}} = \frac{3}{2}$ insulator", Physical Review B **103**, 104401 (2021).

[7]S. V. Streltsov and D. I. Khomskii, "Jahn-Teller Effect and Spin-Orbit Coupling: Friends or Foes?", Physical Review X **10**, 031043 (2020).

[8]S. V. Streltsov, F. V. Temnikov, K. I. Kugel, and D. I. Khomskii, "Interplay of the Jahn-Teller effect and spin-orbit coupling: The case of trigonal vibrations", Physical Review B **105**, 205142 (2022).

[9]N. Iwahara, V. Vieru, and L. F. Chibotaru, "Spin-orbital-lattice entangled states in cubic d^1 double perovskites", Physical Review B **98**, 075138 (2018).

[10]J. P. Allen and G. W. Watson, "Occupation matrix control of d- and f-electron localisations using DFT + U", Physical Chemistry Chemical Physics **16**, 21016–21031 (2014).

[11]A. P. Dioguardi, T. Kissikov, C. H. Lin, K. R. Shirer, M. M. Lawson, H.-J. Grafe, J.-H. Chu, I. R. Fisher, R. M. Fernandes, and N. J. Curro, "Nmr evidence for inhomogeneous nematic fluctuations in $BaFe_2(As_{1-x}P_x)_2$", Phys. Rev. Lett. **116**, 107202 (2016).

[12]D. C. Johnston, "Stretched exponential relaxation arising from a continuous sum of exponential decays", Physical Review B **74**, Publisher: American Physical Society, 184430 (2006).

[13]V. F. Mitrovi ć, M.-H. Julien, C. de Vaulx, M. Horvati ć, C. Berthier, T. Suzuki, and K. Yamada, "Similar glassy features in the ^{139}La nmr response of pure and disordered $La_{1.88}Sr_{0.12}CuO_4$", Phys. Rev. B **78**, 014504 (2008).

[14]A. Abragam, *The Principles of Nuclear Magnetism* (Clarendon Press, 1961).

[15]E. Andrew and D. Tunstall, "Spin-Lattice Relaxation in Imperfect Cubic Crystals and in Non-cubic Crystals", Proceedings of the Physical Society **78**, 1 (1961).

[16]R. Cong, E. Garcia, P. C. Forino, A. Tassetti, G. Allodi, A. P. Reyes, P. M. Tran, P. M. Woodward, C. Franchini, S. Sanna, and V. F. Mitrović, "Effects of charge doping on Mott insulator with strong spin-orbit coupling, $Ba_2Na_{1-x}Ca_xOsO_6$", Physical Review Materials **7**, 084409 (2023).

[17]M. Mehring, *Principles of High Resolution NMR in Solids* (Springer, Berlin, Heidelberg, 1983).
